# Connectedness Is Critical: A Social Network Analysis to Support Emerging Women Leaders in Global Health

**DOI:** 10.5334/aogh.3811

**Published:** 2022-07-28

**Authors:** Angelica Lopez Hernandez, Jennifer L. Weinberg, Amena El-Harakeh, Lola Adeyemi, Neelima Potharaj, Nandini Oomman, Anna Kalbarczyk

**Affiliations:** 1 Johns Hopkins Bloomberg School of Public Health, Baltimore, MD, USA; 2 Department of Nursing and Department of Health Sciences, Monmouth University, West Long Branch, NJ, USA; 3 City University of New York, Graduate School of Public Health and Health Policy, NY, USA; 4 Magna Carta Health, Lagos 101232, Nigeria; 5 Department of Biochemistry, University of Washington, Seattle, WA, USA; 6 Department of Engineering and Technology, University of Washington, Tacoma, WA, USA; 7 The Women’s Storytelling Salon™, Washington, DC, USA

**Keywords:** Social Network Analysis, Women’s Leadership, Global Health

## Abstract

**Background:**

Global health networks serve to bring members together towards a specific objective. However, for myriad reasons, women often lack access to networks that facilitate leadership and career development. In 2020, the Johns Hopkins Center for Global Health launched Emerging Women Leaders in Global Health (EDGE) with a virtual seminar series featuring diverse women leaders followed by an online networking space.

**Objectives:**

The purpose of this paper is to use social network analysis (SNA) to describe the network, the connections within it, and the values placed on those connections to inform future programming.

**Methods:**

We asked EDGE participants to fill out a survey to collect network-specific data. Then, we developed a sociogram and calculated social network metrics based on region, type of organization, and professional career stage.

**Findings:**

The EDGE network had 103 unique connections, and each node, on average, was connected to two other nodes. Early professionals that work in Global North academic institutions were the most prevalent members and most efficiently connected with other members of the network. However, senior professionals from the Global South are key to bridging gaps between regions and across sectors.

**Conclusions:**

Early career professionals from the Global North and senior professionals from the Global South need to work in synergy to improve the connectedness of emerging women leaders around the world.

## Background

Leaders need adequate social capital to function well within organizations and pursue individual development. Social capital theory emphasizes the value of collaboration and knowledge sharing within social networks [1]. Networks are webs of individuals linked by common interests that involve interaction for the purpose of mutual benefits [2]. They allow for social and professional relationships to develop and foster various benefits [3]. Benefits include emotional support in the form of friendships and trust [4], access to contacts who can provide new information, resources, and opportunities, and various career advancements [3].

The power of a network to benefit one of its members is predicated by three interrelated factors: network centrality, closeness, and status [5]. The interaction of these factors influences whether a network can be leveraged effectively by its members to achieve professional goals [2]. Network centrality measures how integral an individual is within the network to which she belongs while closeness and status refer to how close an individual’s relationship is with other network members and the level of influence or seniority those members have within the community.

Within global health, various networks exist that serve to bring together members who produce knowledge, exchange information, advocate, provide funding, develop policy ideas, organize campaigns, and implement programs [6]. Networks focused on various issues within global health have shown to raise attention to and attract resources for high burden health conditions; bring together expertise to solve these problems; and add new voices to processes by giving members a chance to collaborate, contribute, and be heard when they may have otherwise lacked that opportunity [7]. The effectiveness of a network is assessed by looking at the extent to which it can enact change and progress to meet its members’ goals.

Within the workplace or professional field, networks are an important aspect of social capital for career development and progression. The development of interpersonal and professional capabilities that are crucial for effective leaders requires building successful ties within networks. Networks are important for gaining upward mobility, facilitating personal and professional development, making connections, and accomplishing tasks [5] by helping individuals cultivate information exchange, collaboration, alliance development, acquisition of tacit knowledge, visibility, and support [8].

Women often lack access to the social capital that networks provide and which could help facilitate leadership development and career success [2]. This at least partially explains why women are still underrepresented in the top ranks of organizations [5], including those within global health where women hold only 25% of senior leadership positions despite comprising almost 70% of the global health workforce [9]. When used for career development, women’s networks tend to be leveraged for building connections and fostering support [10]. Women-focused networking and training programs produce women who are more likely to aspire to leadership positions [11] and lead with greater confidence, sense of agency, expanded networks, skill development, and self-awareness [12]. In women-only networking groups, women derive a greater sense of psychological safety, which is important for facilitating peer exchange and working collaboratively on solutions [13].

In 2020, the Johns Hopkins Center for Global Health launched an initiative to support Emerging Women Leaders in Global Health (EDGE). The EDGE initiative began with a virtual seminar series featuring diverse women leaders in non-academic careers who shared their leadership stories and key lessons learned [14]. During the seminars and via follow-up emails, attendees were invited to join an online networking space, maintained in Slack, where they could continue to make connections, post questions, and strengthen their networks. Both the seminar series and network are held in virtual spaces and are open to anyone who wants to join. We conducted a social network analysis (SNA) of the EDGE network to describe the network, the connections within it, and the values placed on those connections to inform future programming.

## Methods

### Study Population

All participants of any EDGE activity registered with their full name and email; some provided organizational affiliation, student status, and country, though this data is not complete or consistent across all members who join. For this SNA we recruited any EDGE participant as of November 2020.

### Data collection

To characterize and better understand the relationships among the individuals who participate in EDGE, a sociometric survey was developed based on established network properties [15,16,17] and in collaboration with social network analysis experts. The survey tool began by asking participants to share demographic information including the country they are from, their gender, the type of organization with which they are currently or most recently affiliated (i.e., university, NGO, private industry, multilateral organization, or government), their career stage (i.e., student, trainee, early professional, mid-level professional, or senior professional), and an estimate of how many connections they made through the EDGE programs. Connections were defined as “a shared conversation on LinkedIn, meeting for an informational interview, or a more substantive interaction.” Then, for each connection, participants were asked to provide information about that connection’s country, organization, career stage, and the extent to which they agreed (five-point Likert scale) that the connection helped to advance their career goals in the immediate term.

This survey was developed in Qualtrics, an online survey software, and distributed via email to all participants who had previously registered for any EDGE webinars or joined the Slack network. This email explained the objectives of the research study and stated that participation was voluntary and confidential. Participants had a total of eight weeks to complete the survey, and two reminder emails were sent during this course.

The compiled network data came from event registration forms from the following sources: the Zoom registration forms from the EDGE seminar series held at the Center for Global Health, the two EDGE networking events on the virtual Gather platform, and an attendance sheet from a satellite session from the Consortium of Universities for Global Health conference. Blank responses in the network data database were deleted, and any duplicates by first name, last name, and e-mail address were examined and removed accordingly.

### Data Analysis

Social network analysis is a research methodology grounded in the assessment of empirical data that helps us understand the nature and structure of relationships and interactions within a given community [18]. For the SNA, we developed a sociogram and calculated social network metrics to analyze the relationships among this subset of women working in global health. We created different profiles based on the country of origin, region (belonging to either the Global North or Global South, where Global South includes any low-and middle-income country (LMIC)), type of organization (academia, non-governmental organization (NGO), government, private industry, and multilateral), and professional career stage (trainee, early, mid, or senior).

We calculated centrality measures (degree, closeness, and betweenness), weighting the connections by frequency of interaction to understand key characteristics for information flow [15]. Collectively, these measures locate the primary leaders within a network and the extent to which they engage and broker assets with others. All network visualizations and metrics were obtained using Kumu, an online software designed to facilitate stakeholder, systems, asset, and social network mapping [19].

This research was approved by the Johns Hopkins Bloomberg School of Public Health Institutional Review Board.

## Results

The survey was sent to 1,285 individuals and we received 114 responses, giving a response rate of 8.87%.

SNA was used to illustrate the typology of the relationships within the network. In total, the network had 103 unique connections, and each node (representing a type of individual based on their demographic characteristics), on average, was connected to two other nodes. As shown in Table 2, the most prevalent type of organization was academia, (35 nodes), which corresponds to 47% of the network. Further, the number of connections, the reach, and connectedness varied significantly between different types of subgroups (see Table 1). Despite having a similar number of nodes between the Global North and Global South (37 vs. 36), the average number of connections (degree) was higher for members in the Global North compared to those from the Global South (2.7 vs. 1.5).

**Table 1:** SNA Measures, Definitions, and Findings.


MEASURE	DEFINITION	NETWORK MEANING/INTERPRETATION

Centrality	Measure of a node’s overall influence in the network Degree: a node’s number of connections Closeness: a node’s distance to other nodes Betweenness: a node’s frequency of location in the connection between two other nodes	Nodes from the Global North and Global South have a similar number of local connections. Elements with high closeness like senior professionals are more visible and can spread information more easily. Elements from the Global North had a significantly higher betweenness than the Global South, and they act as key bridges/potential bottlenecks.

Eigenvector	A node’s connection to other well-connected nodes	Elements from academia in the Global North had the highest eigenvector, and they act as leaders of the network, however their local influence is low.

Reach	A portion of a network within two steps of an element	There were no significant differences in reach, meaning that nodes are generally more isolated and cannot affect change through friend-of-a-friend contact.


**Table 2:** Characteristics of the EDGE network by participant characteristics.


	REGION OF THE WORLD		TYPE OF ORGANIZATION					CAREER STAGE				WHOLE NETWORK
		
	GLOBAL NORTH	GLOBAL SOUTH	UNIVERSITY/ACADEMIA	MULTILATERAL	NGO	PRIVATE SECTOR	GOVERNMENT	TRAINEE	EARLY PROFESSIONAL	MID PROFESSIONAL	SENIOR PROFESSIONAL	

Size (total number of connections)	37	38	35	8	19	12	8	17	25	30	6	**103**

Degree (average number of individual connections)	2.7	1.5	2.6	0.6	1.7	1.8	2.25	2	2.2	2.1	2.8	**2.1**

Betweenness	0.89	0.44	0.933	0	0.25	0.12	0.14	0.32	0.39	0.59	0	**0.16**

Closeness	0.18	0.16	0.20	0.15	0.16	0.17	0.19	0.18	0.20	0.19	0.21	**0.21**

Eigenvector	0.63	0.29	0.63	0.05	0.13	0.05	0.013	0.26	0.32	0.35	0.05	**0.10**

Reach	0.13	0.11	0.15	0.09	0.10	0.08	0.12	0.13	0.14	0.11	0.15	**0.14**

Value of each connection	2.2	2.3	2.1	2.1	2.3	2.8	2.8	1.0	2.0	2.9	5.0	**2.29**


When considering the type of organization each node belongs to, we found that academia, mid, and early professionals comprise more than half of the nodes in the network. Members from multilateral organizations are less connected (degree: 0.6) compared to NGOs and the private sector (degree: 1.7 and 1.8, respectively) and have a smaller network (8 nodes) compared to NGOs and the private sector (19 and 12 nodes, respectively). Overall, reach is higher in academia (0.15) and government (0.12) when compared to private sector (0.08), which means that women in academia and in government, on average, can more efficiently connect with one another compared with actors in the private sector.

In relation to their professional stage, early- and mid-career professionals were both the most prevalent members (55 nodes), but senior professionals had the highest average number of connections (degree: 2.8) and can spread information to the rest of the network most easily (closeness: 0.21). Women working in academia in the Global North, regardless of their career stage, have a higher likelihood of being leaders of the network (eigenvector; 0.63 for each subgroup). Despite different career stages having a similar exposure (reach between 0.15 and 0.11), members from the Global North and academia are at an advantage as they gain more exposure through each direct relationship (reach: 0.13) when compared to their counterparts from the Global South or from other sectors.

Members from academia and members from the Global North had the highest betweenness (0.93 and 0.89, respectively), which indicates that they enable the flow of information across the network. Multilateral agencies were not fundamental for information flow (betweenness: 0) but had the same ability as other members to spread information and be visible across the network (closeness: 0.15 and reach: 0.09).

In relation to the value of each connection in advancing their career in the short term, the average value was two out of a five-point Likert scale (SD: 1.1) across the network, which means that this virtual initiative created more connections but did not necessarily improve a member’s career in the short term. In addition, this value did not significantly differ by location. However, this value did differ between different sectors where women from government and private sector had the highest value (2.8) while women from academia had the lowest value (2.1). All in all, women from academia in the Global North are better positioned to be leaders of the network because they have more exposure to a wider range of actors and enable the flow of information. However, as mentioned previously, this does not necessarily translate to advancing their careers more if the network is not inclusive of other regions and sectors.

**Figure 1:**
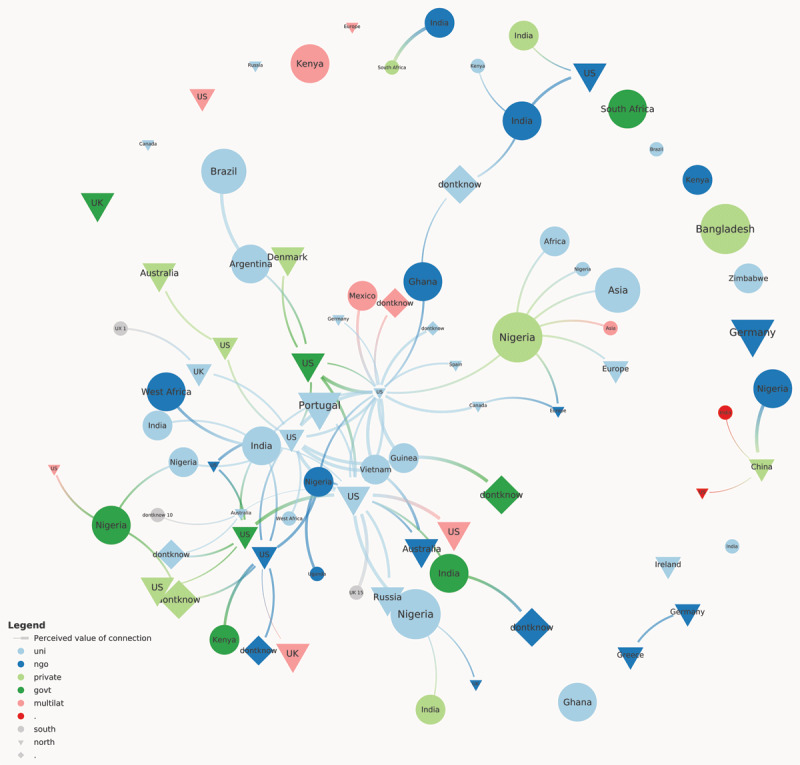
EDGE network’s centrality. Nodes with more influence provide access to decision-makers.

Figure 1 demonstrates how the interrelationships between these subgroups are distributed within the network. The nodes are scaled by the career stage, where senior professionals have the largest nodes. In addition, the width of each connection is scaled by the value each member assigned to the connection. As shown in Figure 1, the primary member of the network was a trainee from the academic sector in the Global North; these types of professionals are responsible for coordinating the network and act as a key bridge connecting other members within the network. More specifically, for this network, the major brokers of connections are from the US and Nigeria. We also found that senior professionals, particularly from the Global South, were key to bridging the gap between regions and across sectors. In addition, senior professionals also tend to connect with other elements from the Global South. Finally, members rated their connection as more valuable when they were connected to a more senior professional from a different sector.

## Discussion

### Overview of Findings

The SNA shows that this network is largely driven by members of the academic sector, particularly from the Global North. Almost half of the members are early-career professionals, which means that this group has the potential to spark global and diverse connections because they have the highest visibility and variety of connections. We also found an interesting interplay between women from different career stages. While fewer senior professionals are represented in the network, on average they have more connections that spread across sectors than do their junior colleagues. This confirms earlier research showing that more senior researchers are well connected [20]. However, early-career professionals in this network are the key **brokers** between members who are not very well connected. That is, they are able to reach across structural holes, bringing un-connected individuals together and increasing the broker’s social capital in the process [21]. Finally, nodes in the periphery did not report any connections, but responded to the survey and were included in the network. It is likely that these nodes are benefitting from other activities in the network.

We also found that while the network was composed of similar numbers of women from countries in the Global North and South, women from the Global North reported a higher number of connections than their counterparts in the South. These connections spanned different sectors and career stages and were key for North-South collaborations. South-South connections were being made but they were seen as less “valuable.” In a study evaluating research capacity building through Global North-Global South networking, respondents from the Global South described partnerships with the Global North as being more important than investing in South–South networks [22]. Senior professionals from the Global South are key to bridging gaps between regions and across sectors, but they also tend to connect with other women from the South. Both nodes on the periphery (nodes with no connections) and the “terminal connections” (terminating nodes) are more likely to be from the South. We know from the literature on mentorship (an important aspect of networking and a key goal for many individuals within various networks) that formal mentoring in LMIC institutions is less frequent and rarely supported by institutions. Most evidence for best practices and norms of mentorship is based on the experiences of high-income countries [23]. This SNA shows that our network has critical gaps for its members in the Global South; the diversity of connections among early professionals in the Global North can and should be leveraged to increase the visibility of members in the Global South.

### Motivations to network

Motivations to network can change as women progress in their careers. As early-career professionals, women need to build networks to identify job opportunities. Once they have found employment, they transition into an invisible mid-career phase for many reasons including structural barriers (workplace structures, human resource biases, inequities), personal, family and lifestyle constraints, and pressure to “just get stuff done” to avoid conflict in the workplace, feel authentic in their roles, and balance work and life demands [24]. The compounding effect of low visibility, low status, low centrality, less power, and lack of access to informal networks (unlike their male counterparts at the same career stage) constrain women’s abilities to build their networks and increase opportunities for leadership development [5].

Unsurprisingly, in our analysis, over half of the network is early- and mid-career professionals from academic institutions. This strongly suggests that women have a strong desire to participate in networks early in their careers compared to women in later stages of their careers, with the need being even greater in the South [25]. While this is not well described in the global health literature per se, recent observations from academic medicine suggest that mid-career is a particularly vulnerable period in women’s career trajectories, when networking and being visible are critical for their career development [26]. Equipping women with skills and opportunities to network while in training and as their careers begin to develop (as is being done in the EDGE initiative) is one way to prepare women early in their careers for leadership positions. The opportunity to exchange ideas, interact with each other, explore collaborations, and find mentors and sponsors not only assists early-career women in identifying employment opportunities but also prevents them from becoming invisible and isolated mid-career professionals.

Our findings show that there is a lack of leadership within this network from women in the Global South. To address this, it is key that South-to-South collaborations are fostered through mentoring, particularly in early career stages and across sectors. There is a special need for professional women communities since research suggests that women-to-women networking provides more effective support and opportunities. Women who form a strong network with other women and share career advice are nearly three times more likely to grow in their careers than women who don’t have this type of support system [27]. More specifically, promoting networks in women’s native languages is a key factor to engage audiences more broadly and to further drive health equity.

Only a few senior professionals participated in the survey suggesting that once individuals are well established in their positions, they may not actively network. However, among those who participated, they did report a higher number of connections and ability to spread information across their networks. This is a lost opportunity for senior professionals (whether in academia or non-academic settings and especially in multilateral institutions) to support and mentor younger women and at the same time learn from them, creating a stronger multigenerational and multisectoral (academia, non-profit, private, and public) network of professional women.

### Tools and approaches to strengthening global networks

Digital platforms like Slack offer the potential to create global networks for women in global health, enabling greater and direct North-South interactions and South-South interactions [28]. This effectively increases the exposure of members from the South at all stages of their careers, though we recognize the unique challenges and limitations of digital tools in LMICs, including regular and reliable internet connectivity. This SNA shows us that we still have work to do to make the network most effective for all its members. To improve global connectivity and enhance the EDGE experience we need to 1) secure and improve connections between members from the Global North and South, 2) improve engagement and connections at regional levels in the Global South, and 3) increase Global South leadership within the network.

Collaboration across global boundaries becomes complex when we consider cultural, temporal, and geographical differences [29]. EDGE’s current use of asynchronous tools such as Slack, along with synchronous events such as live webinars and live mentorship events, provides a strong foundation for overcoming these complexities. For example, the Slack tool enables discourse across time zones eliminating the need to schedule around working hours [30]. However, in its current state, English is the only language currently offered, which may serve as a barrier to engagement. Some topics, generated largely by the EDGE leadership team based in the Global North, may not always be culturally responsive to the pressing needs of members based in the Global South. Therefore, as an initiative through our future synchronous events and asynchronous activities, we seek to prioritize amplifying diverse voices and perspectives, particularly from the Global South.

One way we can achieve this goal is by creating regionally targeted initiatives (i.e., establishment of regional representatives and/or regional network channels with different languages) that may improve the network’s ability to reach individuals in the Global South, further facilitating South-South connections and leveraging senior members’ experience and expertise, thus promoting their leadership within the network. Regional representatives can act as brokers for members from respective regions, helping members navigate the platform and establish new connections. As we saw in our SNA, many global regions have single points connecting them to larger subgroups in the Global North. Identifying and supporting regional representatives may also enhance regional subgroup experiences by further connecting members across boundaries (i.e., South-North). Global South leadership in the network is also critical for the network to remain viable and sustainable. In its current state, a few Global North actors serve as the major brokers—while we do not know with which institutions these actors are affiliated, their absence from the network could have a detrimental effect.

### Multiplier Networking Effect

This analysis of a tightly knit network offers some insights on how powerful this network can be for women in global health to establish direct relationships with peers as well as senior leaders for multiple purposes: increase job opportunities, identify mentors and sponsors, identify potential research collaborators, exchange ideas, and participate in a safe space. This paper paves the way for other institutions to create similar digital networks across sectors and regions. In doing so, a multiplying networking effort would create outcomes at scale not only for women, but to improve health equity more broadly by leveraging its members’ connectedness and expertise in an intersectional manner. Ultimately, increased visibility and ability to connect with others would contribute to greater gender-equitable leadership in global health. In addition, a large-scale network could offer opportunities for further research to understand and document evidence of specific networking purposes and outcomes for women at all ages and stages of their careers.

### Strengths and Limitations

One of the main limitations of this type of analysis is the lack of completeness when assessing the network. Our results are highly dependent on our respondents’ participation, which can predispose to selection bias. We acknowledge that the survey respondents represent ten percent of the total registered EDGE network, for which we do not have complete or consistent demographic data. However, despite the relatively small sample size (114 women), this is most likely representing the more active members in the network that were participating in more than one activity, i.e., the connection brokers. For our findings to be scalable, networks should focus on engaging members that tend to be more passive in their networking efforts to understand how these different networking styles are complementary and fundamental to all network’s wellbeing. The survey tool was only conducted in English which may have reduced participation from members most comfortable in another language.

A strength of this type of analysis is that we accounted for the value and frequency of the connections (our metrics are weighted by betweenness) as well as element-specific attributes like size and reach. These network-specific metrics are meant to be interpreted in relation to the network as a whole and are not meant to be compared between other networks.

## Conclusion

Engaging women at all stages of their careers is critical to promoting more resilient and equitable networks for women leaders globally. We found that early-career professionals from the Global North are the key brokers for facilitating connections between members who are not well connected. Further, these connections were usually facilitated through a senior professional from the Global South. This creates several bottlenecks that could be avoided by promoting regional networks and engaging women at all career stages.
